# Supplementary material for: Interaction of Soybean (Glycine max (L.) Merr.) Class II ACBPs with MPK2 and SAPK2 Kinases: New Insights into the Regulatory Mechanisms of Plant ACBPs
Source: Plants (Basel). 2024 Apr 19;13(8):1146. doi: 10.3390/plants13081146 (PMC11055065; doi:10.3390/plants13081146)
Supplement: Supplementary file 1 [file plants-13-01146-s001.zip › plants-2941173-supplementary.pdf]

Table S1. Sequences of primers for generation Y2H, BiFC and *in vitro* kinase assay constructs.

Name	Nucleotide sequence from 5´ to 3´ [a]	Orientation	Use
**ML3564**	CCGGAATTCTCCTTCAAAGACGACAATCTC (*Eco*RI)	Forward	In generation of plasmids pAT1103, pAT1114
**ML3565**	CGCGGATCCTCAATTTACTTCTCCCCCCACA (*Bam*HI)	Reverse	In generation of plasmid pAT1103
**ML3566**	CCGGAATTCTCCTTCAAAGACGACAATCTC (*Eco*RI)	Forward	In generation of plasmids pAT1104, pAT1115
**ML3567**	CGCGGATCCTCAATTTACTTCTTCCCCCACA3 (*Bam*HI)	Reverse	In generation of plasmid pAT1104
**ML3568**	CCCCCCGGGGATGTACAGAGAGCGAGGAGG (*Xma*I)	Forward	In generation of plasmid pAT1096
**ML3569**	CCGCTCGAGTCAGGGCTTGTGAAGTTTGA (*Xho*I)	Reverse	In generation of plasmid pAT1096
**ML3570**	CCGGAATTCATGCAGAAGCATGGTTTT (*Eco*RI)	Forward	In generation of plasmid pAT1097
**ML3571**	CCGCTCGAGTCAAGAAGAGTCTTTGTCTTT (*Xho*I)	Reverse	In generation of plasmid pAT1097
**ML3572**	CCGGAATTCATGGAAGGAGGAGGAGCTG (*Eco*RI)	Forward	In generation of plasmid pAT1098
**ML3573**	CCGCTCGAGCTACTGCTGATACTCAGGGTTAAA (*Xho*I)	Reverse	In generation of plasmid pAT1098
**ML3574**	CGCGGATCCCGATGGAAAACAATGGTGCTGA (*Bam*HI)	Forward	In generation of plasmid pAT1099
**ML3575**	CCGCTCGAGCTATTCCAACATCTGATGATCCT (*Xho*I)	Reverse	In generation of plasmid pAT1099
**ML3576**	CCGGAATTCATGGAGCGCTATGAGATCCT (*Eco*RI)	Forward	In generation of plasmid pAT1100
**ML3577**	CCGCTCGAGTTAAATAGGACACACAAATTCAC (*Xho*I)	Reverse	In generation of plasmid pAT1100
**ML3578**	CGCGGATCCCGATGGAACGGTATGAGAT (*Bam*HI)	Forward	In generation of plasmid pAT1101
**ML3579**	CCGCTCGAGTCACAATGCACAGACAA (*Xho*I)	Reverse	In generation of plasmid pAT1101
**ML3580**	CCGGAATTCATGGATCGGGCGGCGTTGAC (*Eco*RI)	Forward	In generation of plasmid pAT1102
**ML3581**	CCGCTCGAGCTATATGGCATACACTATCTC (*Xho*I)	Reverse	In generation of plasmid pAT1102
**ML3608**	CGCGGATCCTTCCATTTGTGAGTCACTGC (*Bam*HI)	Reverse	In generation of plasmid pAT1114
**ML3609**	CCGGAATTCGCCATTCATGGATTTGCCAG (*Eco*RI)	Forward	In generation of plasmid pAT1116
**ML3610**	CGCGGATCCGTTTGACTCACAGATGTCACG (*Bam*HI)	Reverse	In generation of plasmid pAT1116
**ML3611**	CCGTCTAGAATGGAAGGAGGAGGAGCTG (*Xba*I)	Forward	In generation of plasmid pAT1119
**ML3612**	CGCGGATCCCGGGCCCTACTGCTGATACTCAGGGTTAA (*Bam*HI)	Reverse	In generation of plasmid pAT1119
**ML3613**	CGCGGATCCATGGAACGGTATGAGAT (*Bam*HI)	Forward	In generation of plasmid pAT1120
**ML3614**	CCGCTCGAGCGGGCCTCACAATGCACAgACAA (*Xho*I)	Reverse	In generation of plasmid pAT1120
**ML3615**	CCGCTCGAGATGGAAGGAGGAGGAGCTG (*Xho*I)	Forward	In generation of plasmid pAT1121
**ML3616**	CGCGAATTCCTACTGCTGATACTCAGGGTTAAA (*Eco*RI)	Reverse	In generation of plasmid pAT1121
**ML3617**	CGCCTCGAGATGGAACGGTATGAGAT (*Xho*I)	Forward	In generation of plasmid pAT1122
**ML3618**	CGCCTCGAGATGGAACGGTATGAGAT (*Xho*I)	Reverse	In generation of plasmid pAT1122
**ML3150**	CTCGAGGACGACAATCTCACCGTCAC (*Xho*I)	Forward	In generation of plasmid pAT963
**ML3129**	TGGGGGAAGAAGTAAATTGACTCGAG (*Xho*I)	Reverse	In generation of plasmid pAT963

[a] Restriction sites are underlined and shown in parentheses.

Table S2. Kinase-specific phosphorylation sites of Class II GmACBPs predicted by GPS 6.0.

**Phosphosite position**	**Code**	**Phosphopeptide sequence** [a]	**Score**	**Kinase family**
49	T	AAAETTTTTRDDAVS	0.0615	CK
57	S	TRDDAVSSDARPFEE	0.3391	CK
78	S	HGSVRNDSDGDYDDD	0.4620	CK
120	S	RLSQKVSSDVQLQLY	0.0784	CK
140	T	ATEGPCSTPQPSPLK	0.2057	CK
179	T	YIDIVTETYPTWLDG	0.0536	AGC
182	T	IVTETYPTWLDGSSL	0.0533	AGC
193	S	GSSLRNKSGDSGGHG	0.3440	CK
250	S	KCIENGVSMNLKDSE	0.0910	CK
256	S	VSMNLKDSEGRTPLH	0.1242	CK

[a] Phosphorylation sites are in red.

Table S3. **Kinase-specific phosphorylation sites of Class II GmACBPs predicted by EPSD 1.0.**

**Phosphosite position**	**Code**	**Phosphopeptide** sequence [a]	**Score**	**Kinase family**
56	S	TTRDDAVSSDARPFE	0.007	AGC/CK
57	S	TRDDAVSSDARPFEE	0.007	CK
66	S	ARPFEEESMVAEHGS	0.997	CK
73	S	SMVAEHGSVRNDSDG	1	AGC
78	S	HGSVRNDSDGDYDDD	1	CK/AGC

[a] Phosphorylation sites are in red.

Table S4. Kinase-specific phosphorylation sites of Class II GmACBPs predicted by NetPhos 3.1.

Phosphosite position	Code	Phosphopeptide sequence [a]	Score	Kinase family
18	S	GLIFSYLLA	0.539	CLK
30	S	SIVVSFKDD	0.707	AGC
37	T	DDNLTVTRA	0.438	AGC
49	T	ETTTTTRDD	0.5	AGC
56	S	DDAVSSDAR	0.616/0.576	AGC /CK
57	S	DAVSSDARP	0.566	CK
66	S	FEEESMVAE	0.509	CK
78	S	VRNDSDGDY	0.583	AGC
92	S	EGVESTELD	0.689	CK
93	T	GVESTELDE	0.625	CK
115	S	ADRLSQKVS	0.822/0.665	AGC /PIKK
120	S	QKVSSDVQL	0.625	CK
140	T	GPCSTPQPS	0.588/0.585/0.502	CDK/MAPK/GSK
144	S	TPQPSPLKM	0.642/0.5	CDK/ GSK
149	T	PLKMTARAK	0.779	AGC
187	S	WLDGSSLRN	0.697	AGC
188	S	LDGSSLRNK	0.803	AGC
193	S	LRNKSGDSG	0.515	AGC
213	T	PVFSTFVYE	0.553	CK
222	S	EEYGSDSQM	0.644/0.515	CK/CLK
224	S	YGSDSQMEA	0.633/0.632	CK/PIKK
256	S	NLKDSEGRT	0.995	CK
293	T	NDGQTPLHY	0.501	MAPK
319	S	ADIYSKDND	0.525	CK

[a] Phosphorylation sites are in red.

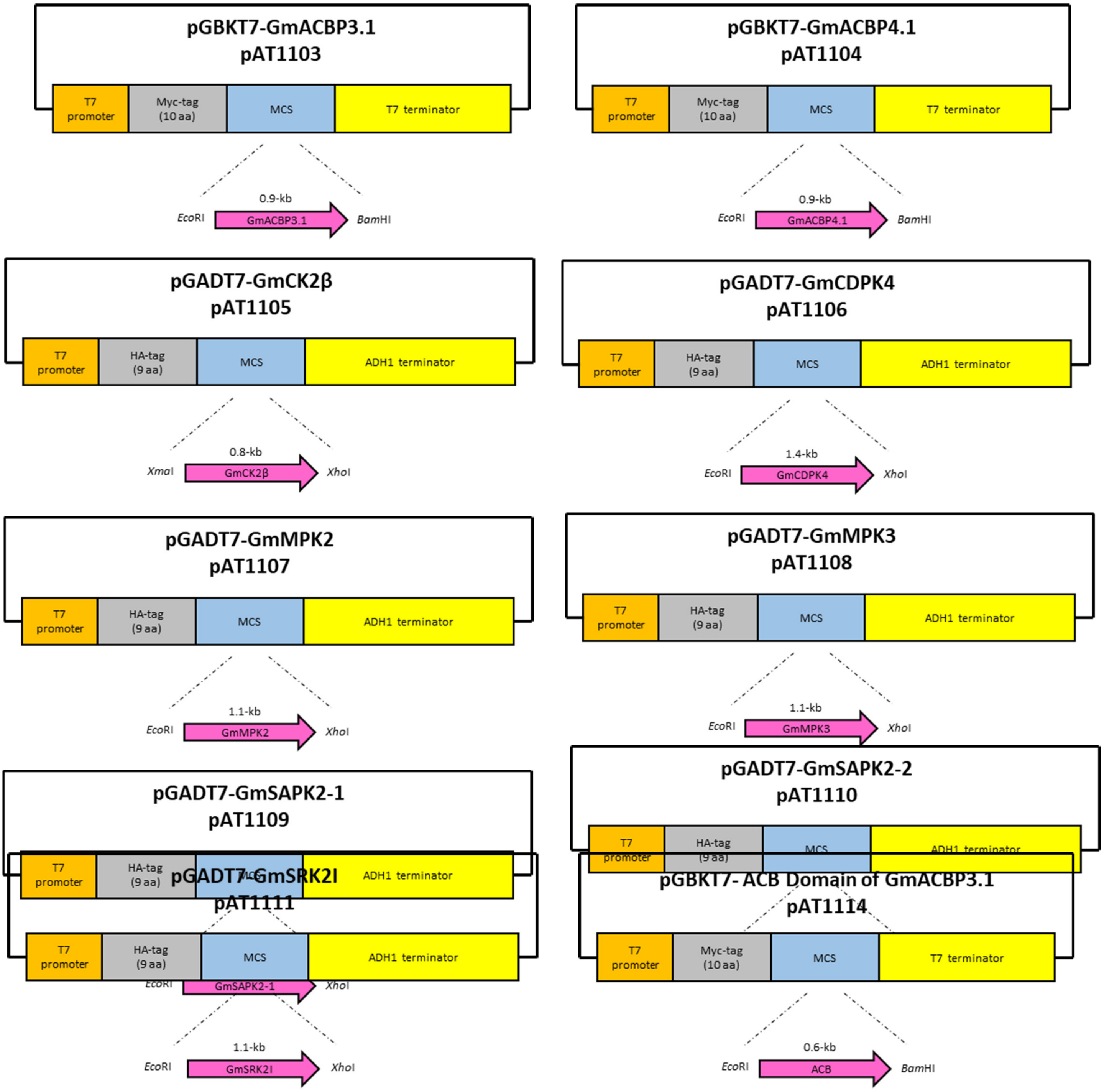
pGBKT7-GmACBP3.1
pAT1103
T7 promoter
Myc-tag (10 aa)
MCS
T7 terminator
0.9-kb
EcoRI
GmACBP3.1
BamHI
pGBKT7-GmACBP4.1
pAT1104
T7 promoter
Myc-tag (10 aa)
MCS
T7 terminator
0.9-kb
EcoRI
GmACBP4.1
BamHI
pGADT7-GmCK2β
pAT1105
T7 promoter
HA-tag (9 aa)
MCS
ADH1 terminator
0.8-kb
XmaI
GmCK2β
XhoI
pGADT7-GmCDPK4
pAT1106
T7 promoter
HA-tag (9 aa)
MCS
ADH1 terminator
1.4-kb
EcoRI
GmCDPK4
XhoI
pGADT7-GmMPK2
pAT1107
T7 promoter
HA-tag (9 aa)
MCS
ADH1 terminator
1.1-kb
EcoRI
GmMPK2
XhoI
pGADT7-GmMPK3
pAT1108
T7 promoter
HA-tag (9 aa)
MCS
ADH1 terminator
1.1-kb
EcoRI
GmMPK3
XhoI
pGADT7-GmSAPK2-1
pAT1109
T7 promoter
HA-tag (9 aa)
ADH1 terminator
GmSAPK2-1
XhoI
pGADT7-GmSAPK2-2
pAT1110
T7 promoter
HA-tag (9 aa)
pGADT7-GmSRK2I
pAT1111
T7 promoter
HA-tag (9 aa)
MCS
ADH1 terminator
1.1-kb
EcoRI
GmSRK2I
XhoI
pGBKT7- ACB Domain of GmACBP3.1
pAT1114
T7 promoter
Myc-tag (10 aa)
MCS
T7 terminator
0.6-kb
EcoRI
ACB
BamHI

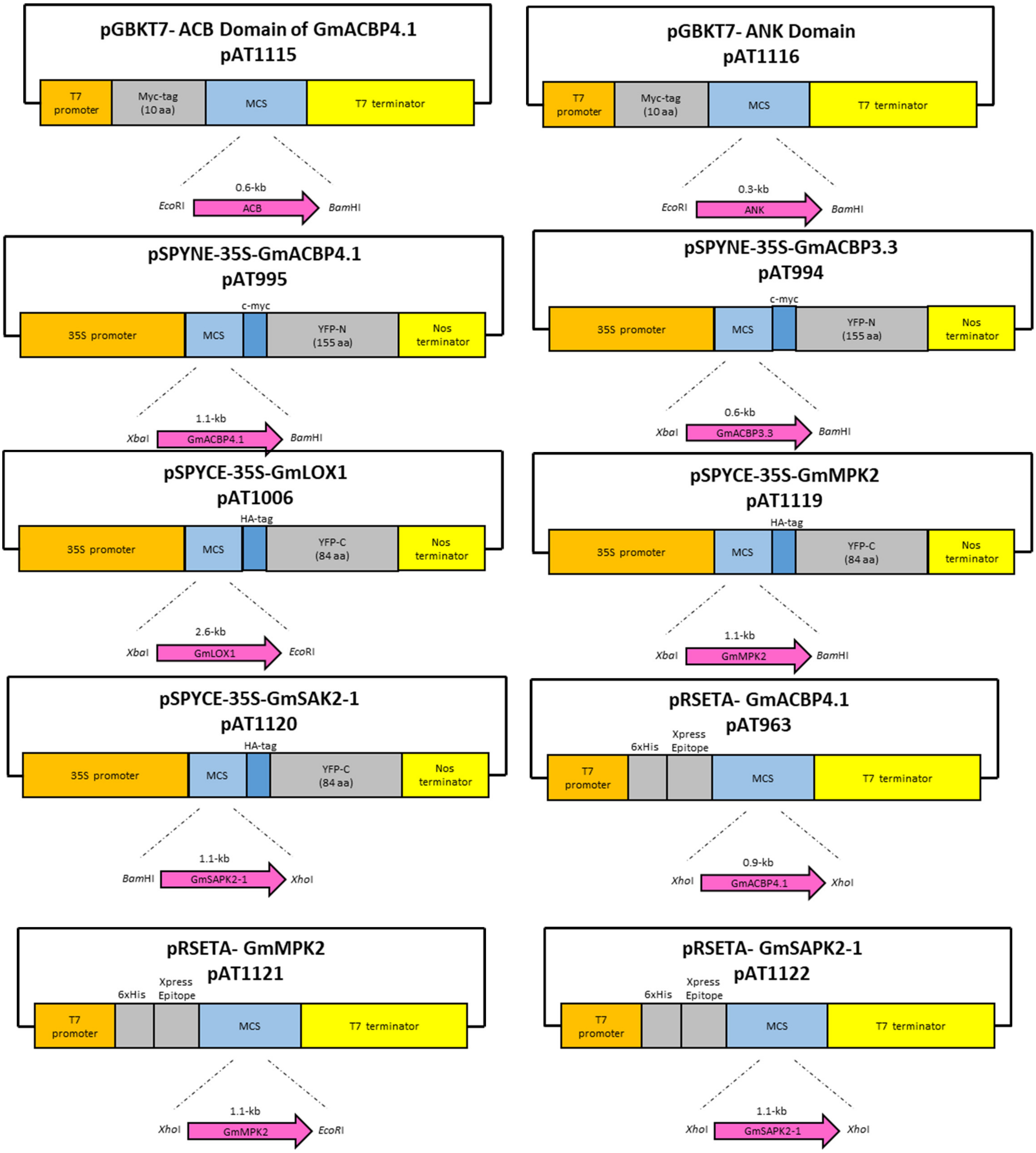

Figure S1. This figure represents all constructs used in this study, including Y2H, BiFC and *in vitro* kinase assays.
